# Analog Accessibility Score (AAscore) for Rational Compound Selection

**DOI:** 10.1021/acs.jcim.4c01691

**Published:** 2024-12-06

**Authors:** Takato Ue, Akinori Sato, Tomoyuki Miyao

**Affiliations:** 1 Graduate School of Science and Technology, Nara Institute of Science and Technology, 8916-5 Takayama-cho, Ikoma, Nara630-0192, Japan; 2 Data Science Center, Nara Institute of Science and Technology, 8916-5 Takayama-cho, Ikoma, Nara630-0192, Japan

## Abstract

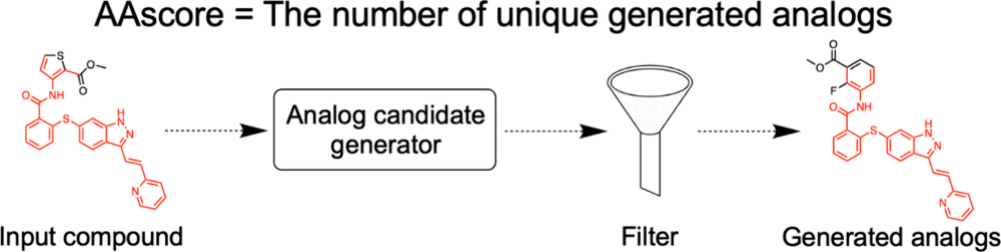

Various *in silico* scores have been proposed to objectively assess the characteristics and properties of a compound. However, there is still no score that represents the analog accessibility of a compound. Such a score would be valuable for selecting compounds proposed by virtual screening or for prioritizing hit compounds for the hit-to-lead phase. This study proposes an analog accessibility score (AAscore), where retrosynthesis prediction and forward product prediction models were utilized to generate virtual analogs. The AAscore is defined as the number of unique analogs and virtual synthetic routes. To evaluate the AAscore in terms of the number of actually synthesized analog compounds, analog compounds were prepared by using the compound-core relationship (CCR) method. It was found that the AAscore was little correlated with the number of CCR-based analogs. Furthermore, AAscores were found to be significantly influenced by the number of extracted candidate reactants from a reactant database. A case study targeting compounds active against carbonic anhydrase 2 showed that the AAscore could identify compounds that were synthesized into analogs.

## Introduction

Synthesizing analog compounds is a common approach in hit-to-lead or lead optimization. In this process, analogous compounds are designed to improve biological activity against the target macromolecule and physicochemical properties to satisfy absorption, distribution, metabolism, excretion, and toxicity (ADMET) requirements. One premise is that these analogous compounds must be synthesized, preferably using a simple and identical reaction scheme. Therefore, when hundreds of diverse hits were presented, prioritizing the hits based on which analogs can be easily synthesized is important for the success of subsequent compound optimization campaigns.^1^ Likewise, in a laboratory, hundreds of virtual compounds identified through computational screening need to be narrowed down for actual synthesis. Testing analog compounds of them will increase the likelihood of finding hits. For this compound selection, a score function representing the synthesizability of analogous compounds could be useful. However, *in-silico* scoring of analog accessibility for a compound is yet to be proposed.

Various *in-silico* scores for a compound have been proposed to objectively assess compound characteristics, including drug-likeness and molecular complexity.^2−4^ Among them, synthetic accessibility has been extensively investigated. For example, the synthetic accessibility score (SAscore) is a score based on a knowledge-based approach that evaluates the synthetic difficulty of a compound based on molecular complexity and fragment contribution.^5^ This score is easy to calculate and is frequently employed with *denovo* molecular generation.^6,7^ The retrosynthetic accessibility score (RAscore)^8^ is a machine-learning (ML) model output of retrosynthesis prediction. The model predicts whether a given compound is virtually synthesized or not. The training compounds for RAscore were collected by conducting retrosynthesis analysis using AiZynthFinder.^9^ The synthetic complexity score (SCscore)^10^ is based on neural network prediction, which was trained on a large number of chemical reactions. Based on the assumption that products are more complex than reactants, the loss function of the network was designed to give a high score for products (complicated molecules). Synthetic Bayesian accessibility (SYBA)^11^ is also based on an ML model of naïve Bayes to understand the contribution of substructures to the score. Furthermore, conventional approaches directly utilizing retrosynthesis-based approaches have been proposed, including retro-score (Rscore),^12^ the retrosynthesis-based assessment of synthetic accessibility (RASA).^13^ The synthetic accessibility proposed by Boda et al.^14^ consisted of structural complexity, similarity to available starting materials, and the frequency of reaction center substructures extracted from a large reaction database.

With rapidly increasing reaction data in combination with the development of sophisticated ML techniques, the prediction ability of data-driven approaches for retrosynthesis analysis has improved.^15−17^ As modeling methods, chemical language-based models, most of which use SMILES strings as input and output, seem promising because they predict reactants in a single-step (end-to-end manner), and also show relatively high predictive ability as supported by several reports.^17,18^ Recently proposed scores for retrosynthetic accessibility, such as RAscore, depend on chemical language-based retrosynthesis analysis tools for its derivation.

In this study, we propose an analog accessibility score (AAscore) to represent the accessibility of analog compounds for an input compound. AAscore is defined as the number of virtual analog compounds by applying a single-step retrosynthesis and forward synthesis prediction after replacing a reactant. The synthesis prediction models can be previously proposed neural network models, and replacement reactants are extracted from a database of purchasable compounds. This simple approach can also provide reaction paths to the analogs, helping to determine which compounds should be prioritized to expand to analog compounds. Situations, in which AAscore can be used, include prioritizing candidate virtual compounds screened by computational methods for synthesis for further experimental testing, and selecting hit compounds for further optimization campaigns if several diverse hits are presented. When the proposed hits are structurally dissimilar, experimental researchers might be interested in the difficulty of expanding to analogs, which becomes a criterion for their selection. It is generally unlikely that most compounds with the same scaffold show the same activity, rather, a small change of a substituent can lead to the opposite activity. Thus, to increase the hit rate, selecting candidate compounds is critical, based on which analog compounds are easy to synthesize. Furthermore, synthesizing analogs is essential to understand the structure–activity relationship. In this phase, an unbiased quantitative score of the number of possibly synthesizable analog compounds with synthesis routes might be helpful. To quantify the analog accessibility with potential reaction paths, we propose AAscore.

## Materials and Methods

### Analog Accessibility Score (AAscore)

**Figure 1:**
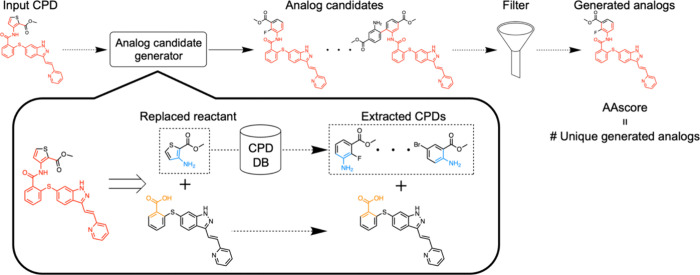
Calculation of the analog accessibility score (AAscore). An input compound is decomposed into potential reactants by single-step retrosynthesis several times. For each reaction, the smallest reactant among the reactants is replaced with compounds from a compound database (CPD DB: the ZINC or eMolecules database was used in this study) based on structural similarity and the reaction center information (blue). Extracted compounds are used with the rest of the reactants for product prediction. The analog candidates are then filtered based on the ratio of the number of heavy atoms in the maximum common substructure (red) to the input compound. The number of uniquely generated analogs is the score for the input compound. Orange represents the reaction center of the larger reactant.

The AAscore is the number of accessible analogs for an input compound by applying single-step retrosynthesis and forward product prediction. One advantage of this scoring scheme is that analogous compounds are actually virtually generated with synthesis routes, assuming the main reactants (unchanged reactants) of the input compound are prepared, making the score transparent. The calculation of the AAscore is described in Figure 1.

To calculate the AAscore, an input compound is first decomposed into sets of potential reactants by applying single-step retrosynthesis prediction trials. The number of retrosynthesis trials is determined based on the accuracy of the retrosynthesis program. Only the retrosynthesis reaction routes that can return to the input compound by forward prediction are regarded as eligible (round-trip validation).^19^

Retrosynthesis prediction for the input compound generates multiple reactant sets. For each reactant set, the smallest reactant in terms of the number of heavy atoms is selected and will be replaced with a compound (candidate reactant) extracted from a database containing purchasable compounds, e.g., ZINC,^20^ while the rest of the reactants being unchanged. The candidate reactants should be similar to the reactant to be replaced and have the same reaction center. In this study, a Tanimoto similarity value of 0.4 was used as a threshold to determine similar reactants (*vide infra*). The reaction centers of candidate reactants or the smallest reactant are identified as follows. First, for each reaction as a result of retrosynthesis, atom mapping was conducted between the reactants and the product by RXNMapper,^21^ followed by the identification of the leaving groups that do not participate in atom mapping. These leaving groups were added to the product side of the reaction, and second-time atom mapping was conducted to identify the reaction centers. Reaction centers were identified by the RDChiral^22^ program. In this study, the radius was set to 2 and “special groups” to “not-use” in the program setting.

Virtual compounds are generated by forward product prediction for the candidate and unchanged reactants. The generated compounds become eligible analogs when they share their scaffolds with the input compound regarding the maximum common substructure (MCS). In this study, the threshold of MCS was set to 2/3 in terms of the number of heavy atoms. Finally, the number of eligible analogs becomes the AAscore. It should be noted that to make analog compounds based on the virtual synthesis routes in the AAscore, the unchanged reactants must be prepared beforehand.

### Retrosynthesis and Forward Product Prediction

For calculating AAscore, in-silico retrosynthesis and forward prediction models are necessary. In this work, we used a fine-tuned T5Chem,^18^ which is a multitask reaction prediction model based on the “Text-to-Text Transfer Transformer (T5)” framework, and a trained LocalRetro,^23^ which is a retrosynthesis framework based on molecular graphs. A T5Chem model can predict both retrosynthesis and forward prediction. Predictive performance using the model for these tasks was reported to be superior to other neural network models.^18^ The input for T5Chem is a SMILES string for retrosynthesis, and a set of SMILES for reactants and reagents for forward product prediction. Reagents were ignored in the forward prediction due to a lack of information in the retrosynthesis output. A publicly available pretrained T5Chem downloaded from https://yzhang.hpc.nyu.edu/T5Chem/index.html was fine-tuned for product prediction and retrosynthesis. A LocalRetro model predicts a set of reactants from a product, i.e. retrosynthesis, which was reported to be superior to other template-based retrosynthesis prediction models.^24^ The input of the LocalRetro model is a molecular graph.

#### Fine-Tuned T5Chem

To fine-tune T5Chem, 260,000 chemical reactions were randomly extracted in the form of reaction SMILES strings from reaction database Pistachio.^25^ This number is about twice the number of reactions used to train T5Chem.^18^

In the fine-tuning phase, the 260,000 reactions were randomly split to 9:1 for training and testing. The training data set was further split into 9:1 for training the network and validation. While the original work of T5Chem focused on five types of reactions, this study used ten types of reactions to make T5Chem compatible with a wider range of chemical reactions. Table S1 in the Supporting Information reports the top 10 reaction classes in the training data set for the T5Chem fine-tuning. The batch size and the number of epochs were set to 64 and 90, respectively. Other parameters were set to default in fine-tuning.

#### LocalRetro

A LocalRetro model was trained on the training data set (both training and validation data) for fine-tuning the *T5Chem* model. After template extraction, the training reactions whose products contained atom templates with a frequency of 5 or less were removed. The training data set consisted of 213,147 reactions. For training the LocalRetro model, the number of epochs was set to 100, and the other hyper-parameters of LocalRetro used the same values as in the original publication.^23^

#### T5Chem and LocalRetro Utilization in AAscore Derivation

Table 1 shows the top-N accuracy for the test data set using the fine-tuned T5Chem model and the trained LocalRetro. For the product prediction task, the top-1 accuracy of fine-tuned T5Chem reached 70%, while for retrosynthesis, 37.7%. Although the LocalRetro model showed higher top-1 retrosynthesis prediction accuracy than T5Chem, for the rest of the top-N accuracies, LocalRetro was inferior to T5Chem. Thus, we mainly present the results using T5Chem in Results and Discussion. In the AAscore derivation scheme, the top 1 product was used. For retrosynthesis, the top 7 reactants among the top 30 predicted reactants were used after a curation process. To ensure the selected reactions were applicable to the input compound, only reactions valid for round-trip prediction by T5Chem were used. Invalid reactants were removed during the curation process. The details of the curation process for reactants are explained in Section S1 of Supporting Information.

**Table 1:** Top-N Accuracy [%] in Retrosynthesis and Forward Synthesis Predictions by the T5Chem and LocalRetro Models

Top-N		1	3	5	7	10	20	30
T5Chem	Product	71.3	84.2	87.5	89.2	90.7	93.0	94.0
	Reactants	37.7	59.0	66.6	70.8	74.6	80.4	83.0
LocalRetro	Reactants	40.0	56.2	61.8	64.6	67.1	70.6	72.1

### Database of Purchasable Compounds

To search for candidate reactants for generating analogs of the input compounds, a database of purchasable compounds is necessary. In this study, two databases were employed: ZINC15^20^ and eMolecules screening compounds.^26^ From the ZINC15 database, compounds were downloaded under the *Anodyne* and *In Stock* conditions, resulting in 10,880,388 compounds. Duplicate compounds were removed based on the canonical nonisomeric SMILES strings. The remaining 7,423,206 compounds were further filtered by eliminating compounds with more than 40 heavy atoms, leading to 7,264,039 compounds. These compounds were used as a database of purchasable compounds in this study. A total number of 18,177,461 screening compounds from eMolecules in the SDF format were subjected to the removal of duplicates based on canonical SMILES strings, resulting in 15,292,006 compounds. Further curation process was applied to this compound data set: duplicates on the basis of nonisomeric SMILES strings were excluded, compounds that could not be handled by an RDKit module and those whose molecular weights were greater than 250 were removed, as processed in a study of retrosynthesis.^9^ The eMolecules database size was 2,005,748, which was much smaller than the ZINC database.

### AAscore Evaluation

AAscore is transparent in terms of virtual reaction paths to analog compounds. To see whether or not the score is correlated with the number of existing analogs for an input compound, additional data sets of analogous compounds were prepared. From the ChEMBL database ver.31,^27^ 1,877,991 compounds were grouped into analog series using the compound-core relationship (CCR) method,^28^ resulting in 241,736 analog series with various scaffolds. From the analog series, 100 diverse series were selected based on similarity calculation using extended connectivity fingerprints with a radius of 2 (ECFP4),^29^ starting from the core with the highest number of analogs, selecting one by one so that similarity values between each pair of cores (scaffolds) were less than 0.2 in terms of Tanimoto similarity. The number of analog compounds per series ranged from 40 to 433, with an average number of 88.

From each of the selected 100 analog series, 10 diverse analogs were selected based on the MaxMin algorithm.^30^ The reported AAscore for a series in this study is the average AAscore of the 10 analogs. In this study, the results using the ZINC database are mainly reported as the compound database from which candidate reactants were selected. The results using the eMolecules database are reported in the Supporting Information.

## Results and Discussion

### Number of Generated Analogs

Ten analogs were extracted from each of the 100 analog series from the ChEMBL database, and AAscore was calculated for a total of 1,000 compounds (AAscore evaluation**section**). These 1,000 compounds were put in the T5Chem-based retrosynthesis model, and a total of 30,000 predicted reactant sets were obtained as top-30. A curation process for the predicted reactants resulted in 4,381 reactant sets from the initial 30,000 outputs. Among the 4,381 reactant sets, 1,922 sets found qualified candidate reactants in the ZINC database that will replace the smallest reactants of the reaction sets. A forward product prediction was conducted for each combination of a candidate reactant and the unchanged reactants, generating 212,516 predicted products. The final curation process selected a total number of 202,798 products (analog candidates). The MCS-based scaffold filter narrowed down this number to 139,466 eligible analogs. The details of the curation process are reported in Supporting Information.

### AAscore Distributions

**Figure 2:**
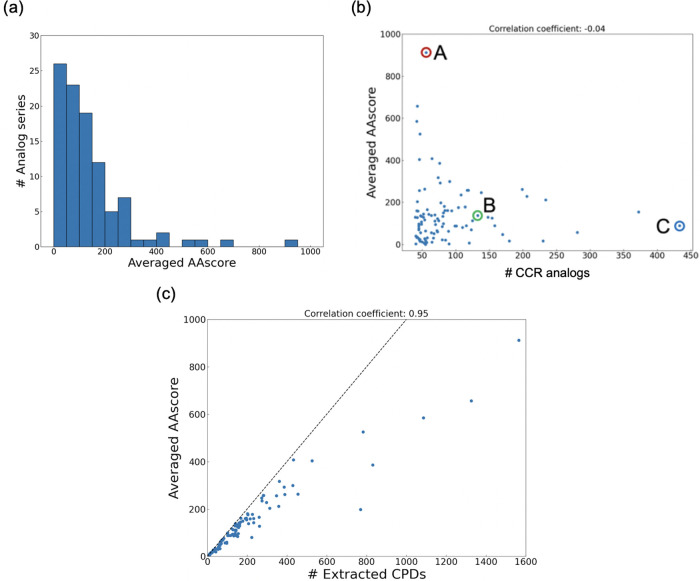
**Distribution of the AAscore for the ChEMBL analog series data sets.** For each analog series, the average of the AAscore for series compounds is assigned. The distribution of the AAscore is reported (**a**). This average AAscore is also plotted against the number of analogs for the core (**b**) and the number of extracted compounds from the ZINC database (**c**), respectively. Colored circles are three exemplary series with high (red, ID: A), middle (green, B), and low (blue, C) AAscore. Details for the three series are provided in Table 2.

The histogram of the average AAscore for the 100 analog series is shown in Figure 2a. The distribution was like a Poisson distribution, with a maximum average score of 913. The mean average AAscore for 100 analog series was 139, with a median of 109 and a mode of 25. There was no correlation observed between the number of analogs per core and the averaged AAscores (Figure 2b) (correlation coefficient: −0.04). Discussion, including possible reasons for the poor correlation, is provided in the Scope and Limitation of AAscore**section**. There was a quite strong positive correlation between the number of compounds extracted from the ZINC database and the averaged AAscore, with a correlation coefficient of 0.95. This suggested that the extraction procedure of candidate reactants, based on the reaction center and similarity, proposed high-quality compounds as candidate reactants. The forward prediction and the MCS filtering process did not drastically remove generated analogs, in particular when the number of extracted compounds from the ZINC database was less than 200 (Figure 2c). On the other hand, for the series having more than 1,000 extracted compounds, these two steps, contributed to narrowing down the analogs. Three series are circled in red, green, and blue, corresponding to high, middle, and low AAscore, respectively, for discussion later (Figure 2b). The same trend of AAscore distributions and the analysis results were observed when LocalRetro was used as a retrosynthesis model, as shown in Figure S1.

There was a weak negative correlation between the averaged SAscore and the averaged AAscore for the 10 analogs in each selected analog series, with a correlation coefficient of −0.20. This implies that the higher the difficulty of synthesizing an input compound, the harder it is to synthesize analogs from that compound. There was no correlation between the average number of heavy atoms in the selected 10 analogs and the averaged AAscore, with a correlation coefficient of 0.04.

### Effect of Compound Databases on AAscore

**Figure 3:**
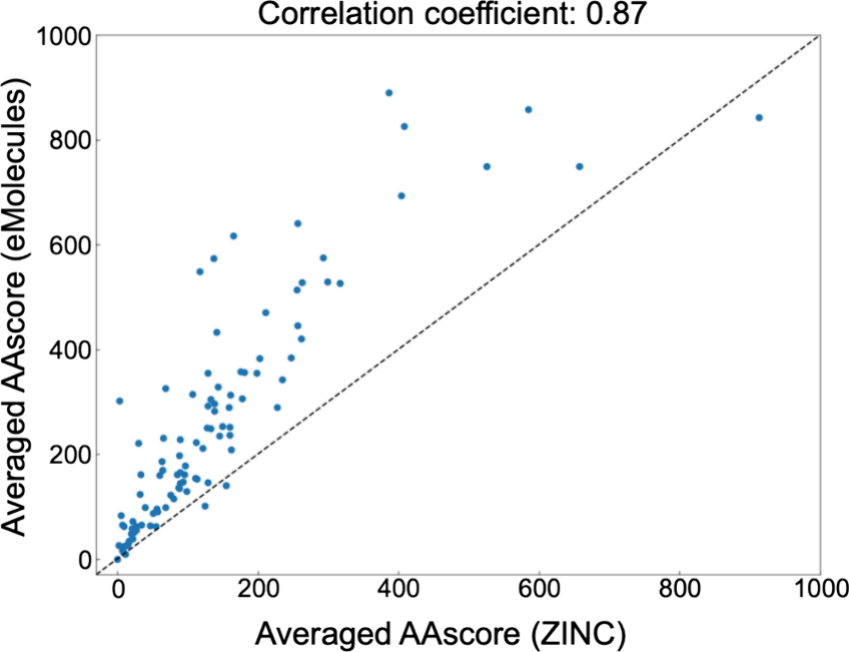
**Effect of the databases of purchasable compounds on averaged AAscore.** The averaged AAscore calculated using the eMolecules database is plotted against the averaged AAscore calculated using the ZINC database.

The proposed AAscore depends on the compound database from which candidate reactants were extracted. In this study, the ZINC and the eMolecules database were compared. AAscores derived by using the eMolecules database were consistently higher than those using the ZINC database (Figure 3), possibly due to using the *Anodyne* condition to make the ZINC database. Using the eMolecules database, among 100 analog series, the highest average AAscore was 890, with an average of 262, a median of 210, and a mode of 75. However, these two scores were highly correlated (Pearson correlation coefficient: 0.87, Kendall rank correlation coefficient: 0.74), and the tendency of the easiness of analog synthesis was preserved. The distributions of AAscores for the eMolecules database are reported in Figure S2, and those with LocalRetro used as a retrosynthesis prediction model are shown in Figures S3 and S4.

### Candidate Reactant Diversity

**Figure 4:**
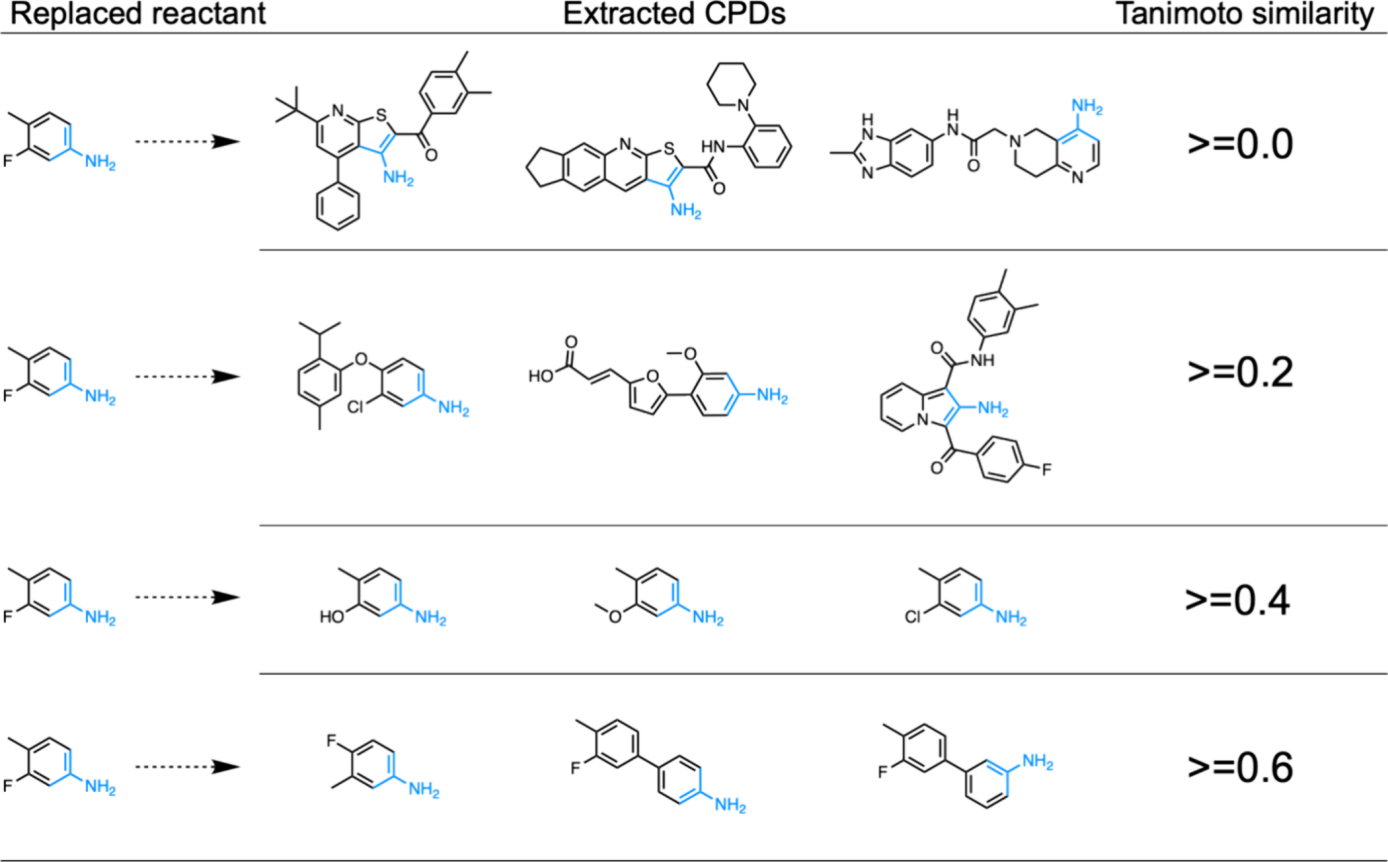
**Examples of extracted compounds for 3-fluoro-4-methylaniline as the replaced reactant at different Tanimoto similarity thresholds.** Blue parts represent the reaction centers of the replaced reactant and the extracted compounds.

Candidate reactants are extracted from a database based on both structural similarity and reaction center matching to the replaced reactant. Systematically identified reaction centers are usually underspecified with respect to the relevant chemical environment and should be extended. Since the atomic environment around the reaction center generally influences the reactivity, neighbor atoms up to a radius of two were also merged into the reaction center. Moreover, a similarity filter was introduced to select relevant candidate reactants. Figure 4 shows examples of extracted compounds for 3-fluoro-4-methylaniline as the replaced reactant at different Tanimoto similarity thresholds from the ZINC database. In this example, the number of extracted compounds was 64,341, 17,606, 293, and 3, corresponding to 0.0, 0.2, 0.4, and 0.6 Tanimoto similarity thresholds, respectively (Figure 4). For generating virtual analog compounds, similar-sized compounds to the replaced reactant should be selected. Based on structural validity and the number of extracted compounds, we determined that Tanimoto similarity between candidate compounds and the replaced reactants should be greater than 0.4. Therefore, extracted compounds containing the extended reaction center and exhibiting a Tanimoto similarity of 0.4 or more to the replaced reactant were used as qualified candidate reactants.

### AAscores for Exemplary Compounds

#### Three Exemplary Analog Series

**Figure 5:**
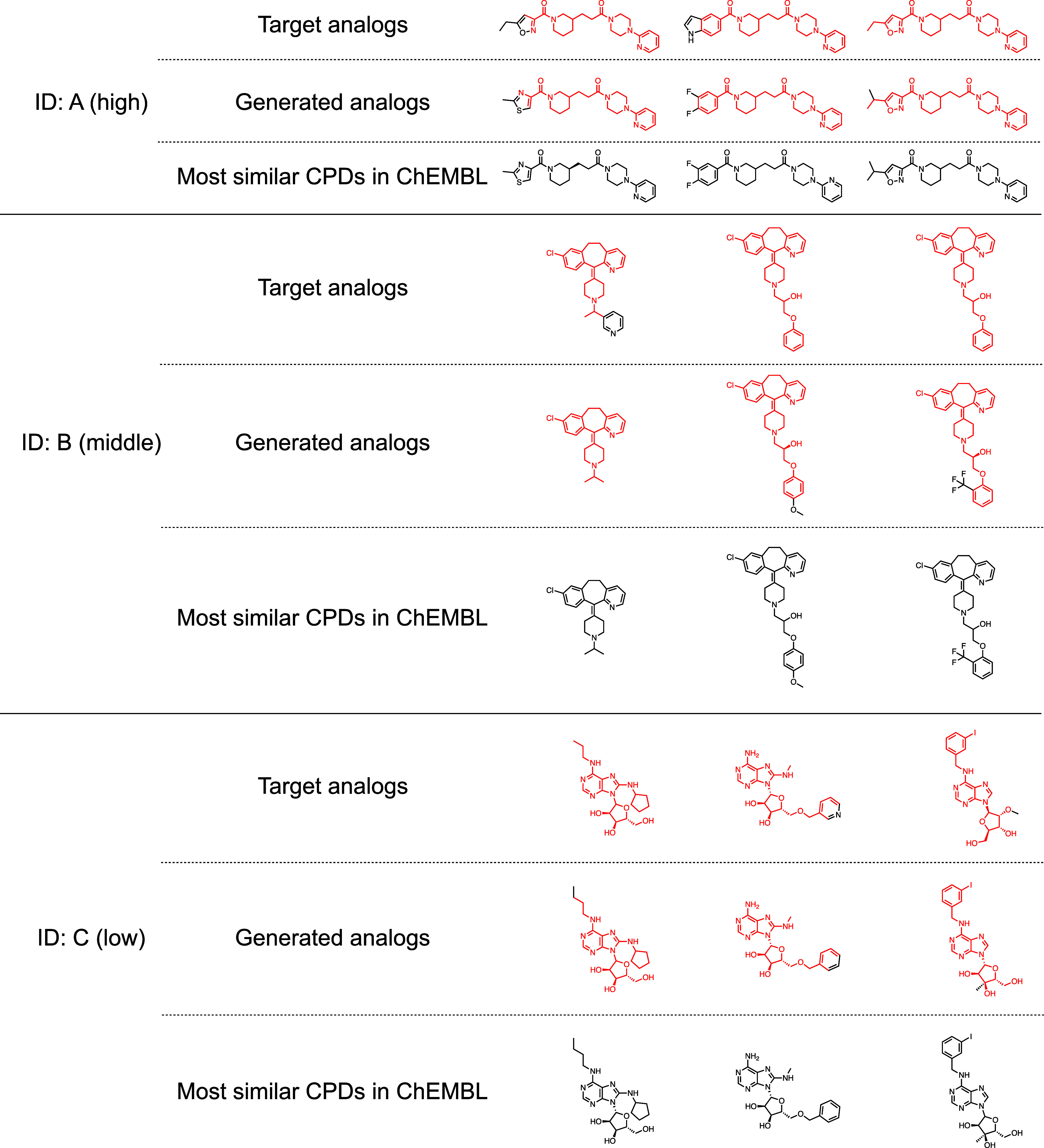
**The top 3 generated analogs with the highest similarity to the compounds in ChEMBL for the three exemplary analog series.** Red represents the maximum common substructure between the target analogs and generated analogs.

Table 2 reports score profiles for the three exemplary analog series shown in Figure 2b. It can be seen from this table that the higher the number of candidate reactants, the higher the AAscore is. After the curation of predicted reactants, only 4, 2, and 1 reactions remained, corresponding to high, middle, and low AAscore, respectively. The number of candidate reactants tended to increase as the number of reactions in the single-step retrosynthesis increased. Among these analog series, the series with the lowest AAscore showed the highest conversion rate to analogs from the candidate reactants. For these three series, the order of actual CCR analogs and AAscores were completely opposite. The core with middle analog accessibility contained a tricyclic ring, which is often found in antidepressant drugs. The core with low analog accessibility is adenosine, a biological molecule whose derivatives (analogs) have been extensively investigated. This bias may be one of the reasons why no correlation between the AAscore and the number of CCR analogs was observed, as shown in Figure 2b. For each analog series in Table 2, the three qualified analogs exhibiting the highest similarity to the ChEMBL compounds are shown in Figure 5. The similarity of all these generated analogs to the most similar ChEMBL compounds was 1.0. The mean maximum similarity between generated analogs and compounds in ChEMBL was 0.69, 0.76, and 0.77 for high, middle, and low analog accessibility, respectively. This indicates that the generated analogs are highly similar to the existing compounds. However, these compounds were not grouped together according to the CCR criterion.

**Table 2:**
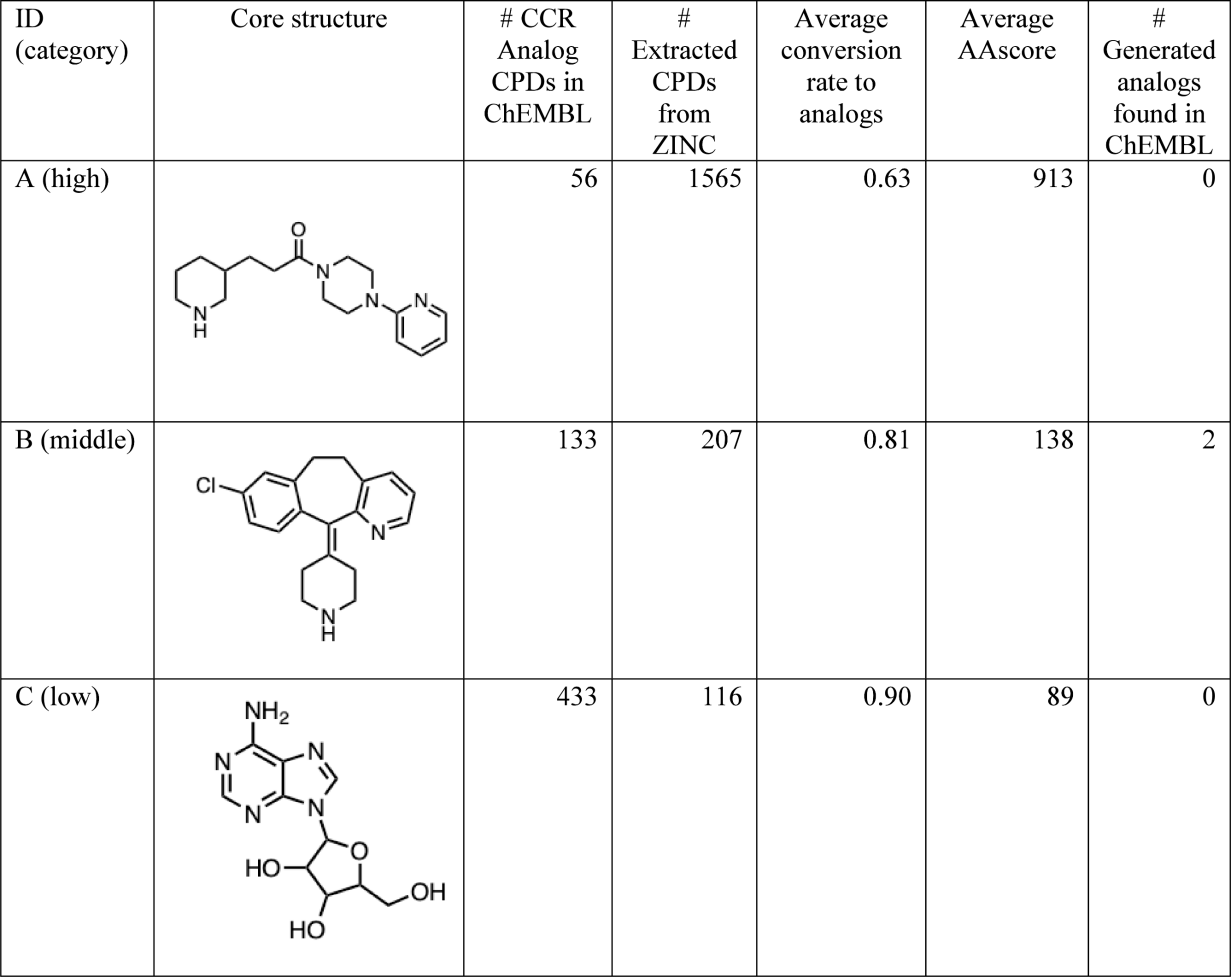
Score Profiles for the Three Exemplary Analog Series.^a^

a For each series, analog accessibility, core structure, number of CCR-based analogs in ChEMBL, number of extracted compounds from ZINC, average conversion rate to analogs, average AAscore, number of generated analogs in ChEMBL are listed.

#### Synthetic Route Examples

**Figure 6:**
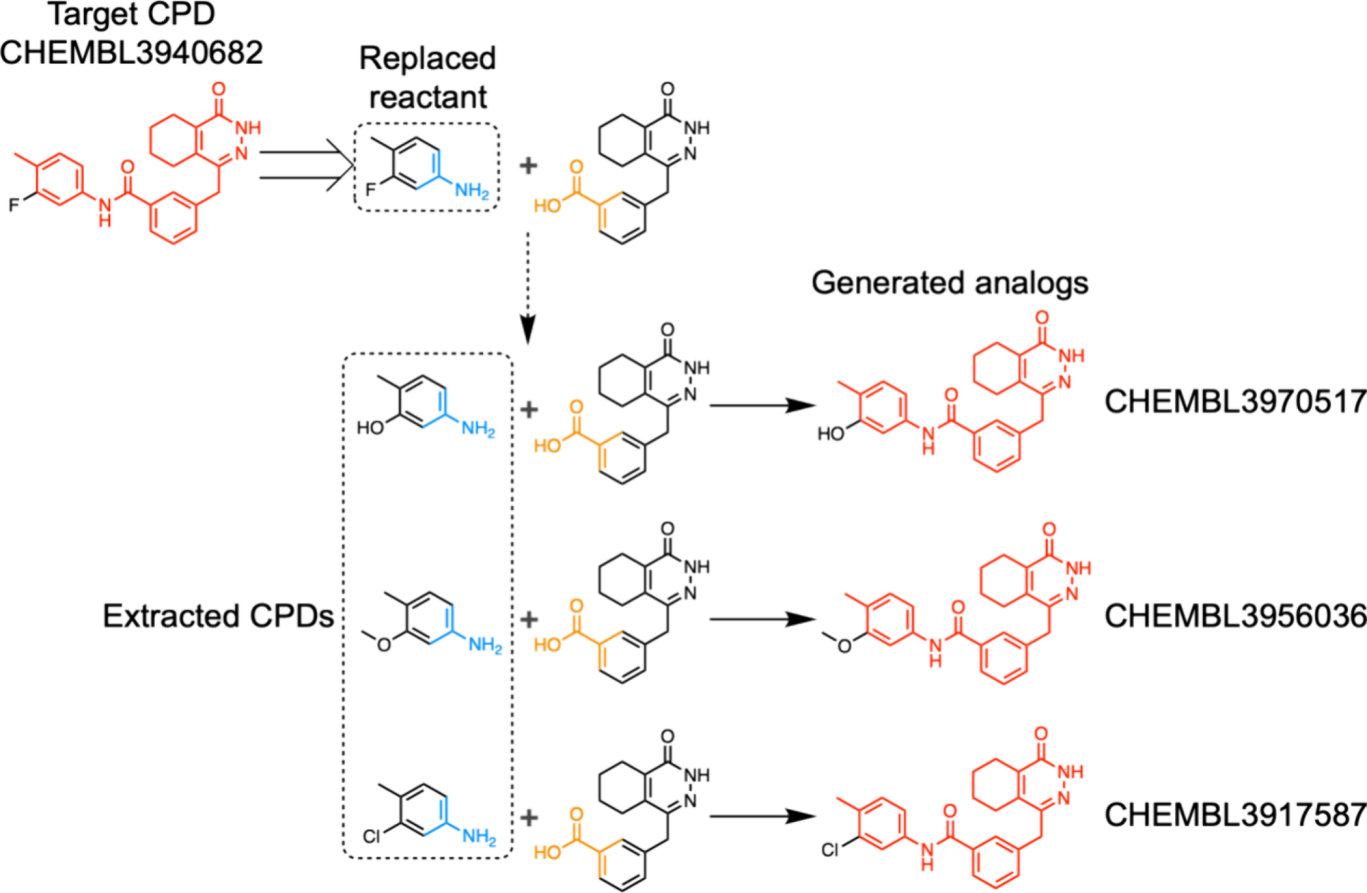
**Example of synthetic routes for generated analogs found in ChEMBL database from CHEMBL3940682**. The same reaction mechanism is assumed in single-step retrosynthesis and product prediction. Red represents the maximum common substructure between the input compound and generated analogs. Blue and orange represent the reaction center of the smallest reactant and the larger reactant, respectively. For each analog, the CHEMBL ID is provided.

As an exemplary case, successfully retrieved analogs (not CCR-based analogs) in the ChEMBL database by the AAscore derivation are reported in Figure 6 (the input compound was CHEMBL3940682). The retrosynthesis prediction proposed to break the amide bond to form an amine and carboxylic acid as reactants. The replaced reactant: amine, was replaced by structurally similar candidate reactants with the same reaction center. T5Chem forward prediction by putting combinations of candidate reactants and the remaining reactant: carboxylic acid, generated three analogs, which were found in the ChEMBL database. Although these analogs might be synthesized from different reaction paths, the AAscore for the target compound was derived partially based on the synthetic route.

#### Compounds against a Specific Target Macromolecule

Another example of the AAscore is provided for target-specific CCR-based analog series against carbonic anhydrase 2. The target-specific CCR series were generated using the ChEMBL database, and 5 diverse analog series were selected in the same way as selecting CCR series using the entire ChEMBL database (AAscore evaluation**section**). From each of the selected 5 series, 3 diverse analogs were selected using the MaxMin algorithm. AAscores were calculated for the analogs (a total of 15 compounds). As examples, four active compounds with high and low AAscores from different analog series are presented in Table 3. The upper two compounds (CHEMBL4450666 and CHEMBL3237855) had AAscore values of 591 and 258, respectively, while the bottom two compounds (CHEMBL3342253 and CHEMBL2047796) had AAscore values of 85 and 53, respectively. For CHEMBL3342253, all extracted qualified compounds from the ZINC database generated analogs by the forward product prediction. Analogs in the ChEMBL database were not retrieved for CHEMBL4450666 and CHEMBL3342253. Bioactive analogs were indeed generated via AAscore calculation for CHEMBL3237855 and 2047796. A possible reason for not identifying the bioactive analogs for CHEMBL4450666 and CHEMBL3342253 is that the candidate reactants required to generate the compounds in the ChEMBL database were not extracted from the ZINC database. For CHEMBL4450666, the retrosynthesis prediction proposed to break the amide bond and the C–N bond to form an amine and carboxylic acid, and amine and halogen (Br, I) compounds, as reactant sets, respectively. For CHEMBL3342253, the retrosynthesis prediction proposed to break the S–N bond attached to the ring to form an amine and chlorosulfonyl as reactants. Different criteria for extracting candidate reactants (reactions) could have generated the compounds contained in the ChEMBL database.

**Table 3:**
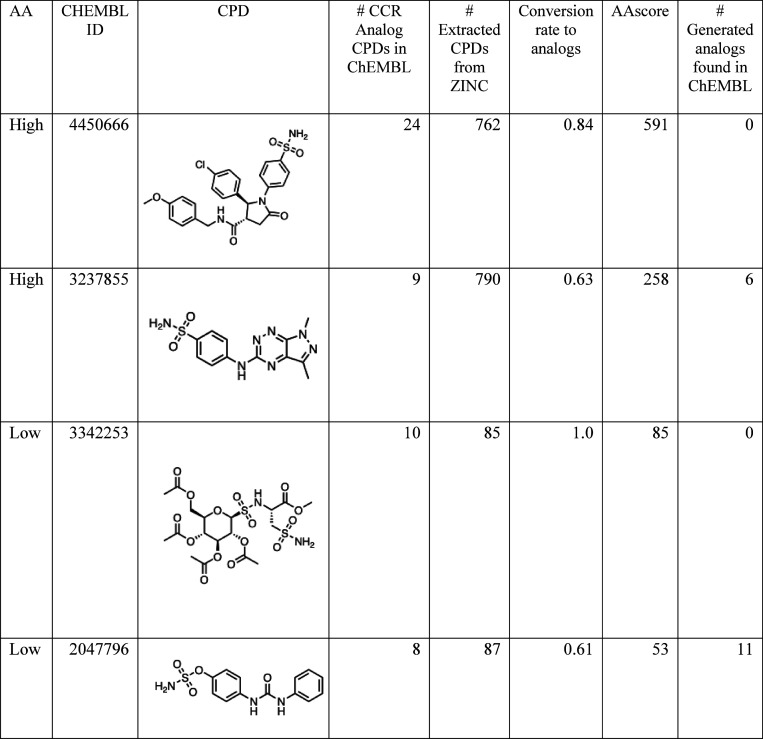
AAscores for Single Target Inhibitors: Carbonic Anhydrase 2.^a^

a For each compound, analog accessibility, CHEMBL ID, compound structure, number of CCR-based analogs in ChEMBL, number of extracted compounds from ZINC, conversion rate to analogs, AAscore, number of generated analogs in ChEMBL are listed.

#### Examples of No-Qualified Candidate Reactants

**Figure 7:**
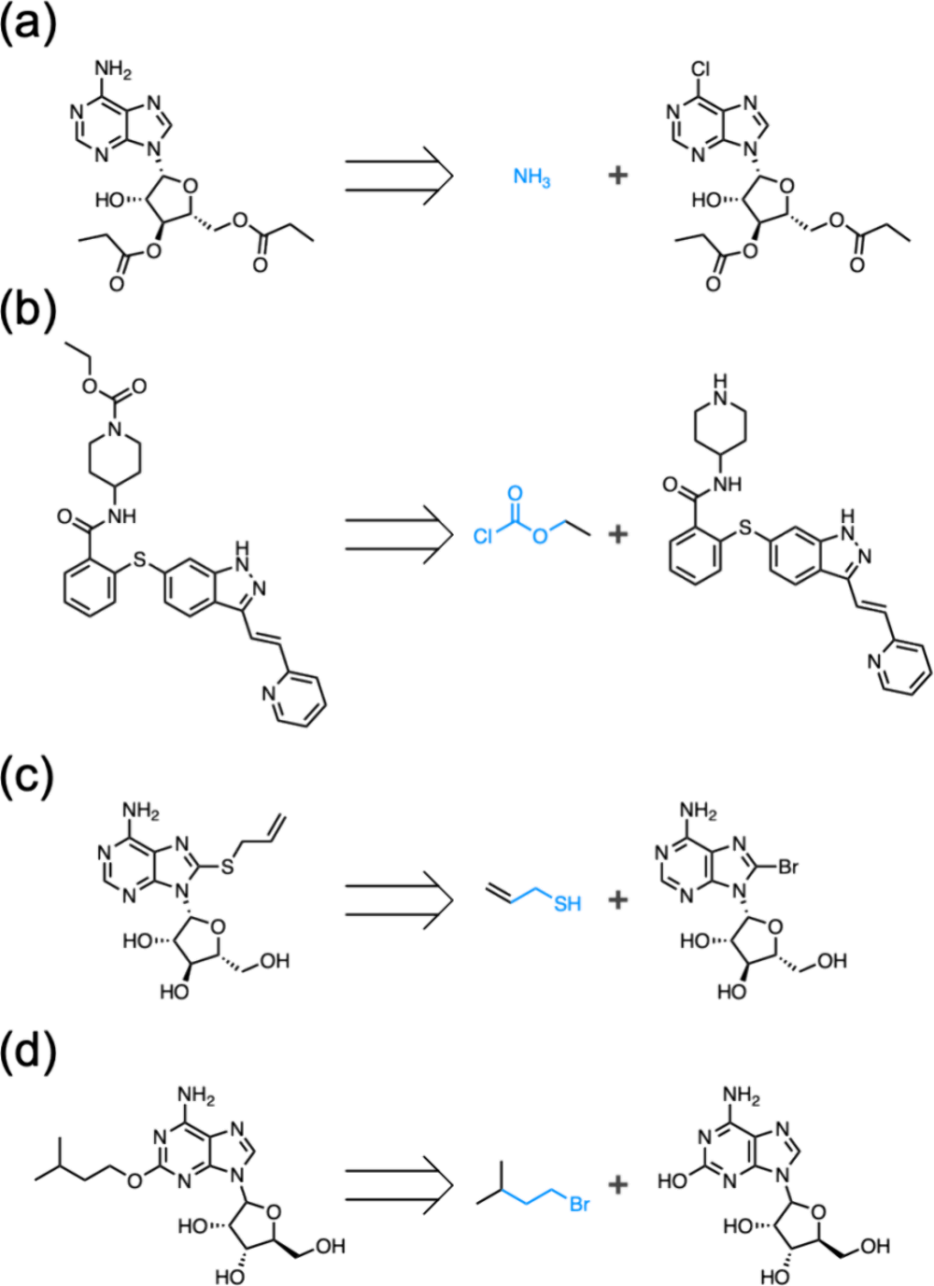
**Examples of retrosynthesis where no qualified candidate reactants are found.** Substructures highlighted in blue represent the reaction centers of the smallest reactants. The reason for not finding candidate reactants is, a single molecule forming the reaction center (**a**), the scarceness of the reaction center pattern (**b**), structurally dissimilar to the reactant (**c**), or both **b** and **c** (**d**).

During the calculation of the AAscore, we encountered a situation where no qualified candidate reactants for a replaced reactant were identified. Four such examples are shown in Figure 7. Figure 7a shows the case where the reaction center of the replaced reactant: ammonia, was ammonia itself. The reaction center, corresponding to the search query, became a single molecule. Figure 7b shows the case where the reaction center of the replaced reactant was uncommon, and no matching compounds were found from the ZINC database. Figure 7c shows the case where the number of heavy atoms of the replaced reactant was low, thus finding compounds with a Tanimoto similarity value of 0.4 was not possible. Figure 7d shows a case where the number of heavy atoms was small, and the reaction center pattern was uncommon. In this case, 142 compounds were found to match the reaction center pattern, while six similar compounds were found using the similarity criterion only.

### Scope and Limitation of AAscore

There are three main areas of improvement and limitations to this study. First, the AAscore relies on the database used for searching candidate reactants and the model’s prediction accuracy. In this study, T5Chem was used to perform forward prediction, and T5chem and LocalRetro were used to perform retrosynthesis prediction. If models with higher prediction accuracy are developed, the AAscore can be calculated in the same way after replacing the models. Regarding the database used for searching candidate reactants, an accessible compound database can be replaced with the ZINC or eMolecules screening databases. Second, the AAscore assumes retrosynthesis with multiple reactants to generate analog molecules. This assumption sometimes contradicts our intuition about analog compounds, particularly when the score takes a zero value. For example, in normal retrosynthesis, benzene is not decomposed into multiple components, and the AAscore for benzene would be zero. However, this does not mean that analogs of benzene do not exist. Third, it takes about 10 min to calculate the AAscore for a single input compound (OS: Windows10, CPU: Intel(R) Xeon(R) W-2295, RAM: 128GB). The most time-consuming part was forward prediction and retrosynthesis prediction using the T5Chem or LocalRetro models. Further speed-up is needed for practical use. Another improvement of the AAscore is to incorporate an option of maintaining an unaltered part of an input molecule. Keeping a specific substructure while generating accessible analogs is sometimes a prerequisite in applications.

In the analysis between the number of CCR analogs and AAscores, our original expectation was that the AAscore would be correlated with the number of analog compounds found in a large compound database, in particular, if the database contains derivatives of bioactive compounds as a result of lead optimization. However, analyses using two retrosynthesis models and two compound databases did not provide positive correlations. We speculated three reasons for this: CCR analogs not containing the analogs AAscore generated (1), the preference of synthesizing analogs of endogenous (natural) ligands and famous drugs (2), and a limitation of AAscore of using a single-step retrosynthesis and forward prediction (3). In point 1, selecting an appropriate definition for analogs was difficult. For example, widely used BM scaffolds^31^ could be used for the analog definition. However, in this case, adding a phenyl ring or replacing a ring atom with another element would generate a different scaffold. Also, fixing a substitution site of the core, like matching molecular series, was not appropriate in this case. The CCR method seemed to be the best for collecting sets of analog compounds with various cores. In Figure 5, for the three input compounds, generated analogs by the AAscore method were actually found in the ChEMBL database. However, they were not categorized into the corresponding analog series. This validation revealed that the AAscore analogs did not cover the whole CCR analogs in the ChEMBL database. The second point is already explained in AAscores for exemplary compounds**section** with an example of adenosine derivatives, and the third point could be overcome. However, using multiple-step synthesis path route prediction would lead to a complicated workflow and more computational cost, which needs further research.

## Conclusions

We have proposed an analog accessibility score (AAscore) to represent the accessibility of analogs for an input compound. The derivation of the AAscore consists of four steps: (1) An input compound is decomposed into potential reactants by single-step retrosynthesis prediction. (2) For each reaction, the smallest reactant is replaced with candidate reactants extracted from the compound database (the ZINC and eMolecules databases in this study). (3) Products are generated from extracted candidate reactants in combination with the rest of the reactants by forward prediction. (4) The unique analogs are obtained by filtering out nonanalog products based on the maximum common substructure analysis. The AAscore is defined as the number of unique analogs generated through these four steps. One of the advantages of this score is its transparency. The score is the number of generated analogs with virtual synthesis routes. Furthermore, components of the AAscore can be fully customized by users: the retrosynthesis and forward prediction models, as well as the compound (reagent) database for selecting candidate reactants, which would be sophisticated as the research in this field proceeds.

To evaluate the AAscore in terms of the number of actually synthesized analog compounds, we prepared analog compounds by using the compound-core relationship (CCR) method. It was found that the AAscores were little correlated with the number of CCR-based analogs. This result suggests that analog compounds in the AAscore were different from CCR analogs and/or extensively investigated analogs were not related to the virtual synthesis. Furthermore, AAscores were found to be significantly influenced by the number of extracted candidate reactants from a reactant database. The proposed extraction process, which selects candidate reactants for the smallest reactant of a reaction based on reaction center and similarity, yielded high quality compounds as candidate reactants. A case study focusing on compounds active against carbonic anhydrase 2 demonstrated that the AAscore could objectively assess the accessibility of analog compounds for an inhibitor for a target. Several improvements in the AAscore are expected, such as reducing calculation time and preparing an appropriate reactant database. With this study as a starting point, we hope that further research and development of indicators and scores to objectively represent the accessibility of analog compounds for a compound will be undertaken.

## Data Availability

Pistachio is a commercial database. The ZINC and eMolecules databases are available from web sites. The script of calculating AAscore is provided in the GitHub repository: https://github.com/U-T100/AAscore.

## Supporting Information Available

Curation procedure (Section S1); reaction classes in the training data for training retrosynthesis and forward prediction models (Table S1); distributions of AAscore using the ZINC database and LocalRetro, eMolecules database and T5Chem, and eMolecules database and LocalRetro (Figures S1–S3); AAscore comparison between the eMolecules and ZINC databases using LocalRetro (Figure S4) (PDF)
