# Supplementary material for: Analog Accessibility Score (AAscore) for Rational Compound Selection
Source: J Chem Inf Model. 2024 Dec 6;64(24):9350–60. doi: 10.1021/acs.jcim.4c01691 (PMC11686423; doi:10.1021/acs.jcim.4c01691)
Supplement: Supplementary file 1 — ci4c01691_si_001.pdf [file ci4c01691_si_001.pdf]

Supporting Information

Analog Accessibility Score (AAscore) for Rational Compound Selection

Takato Ue,[1] Akinori Sato,[2,1] Tomoyuki Miyao[2,1]*

[1]Graduate School of Science and Technology, Nara Institute of Science and Technology, 8916-5 Takayama-cho, Ikoma, Nara, 630-0192, Japan

[2]Data Science Center, Nara Institute of Science and Technology, 8916-5 Takayama-cho, Ikoma, Nara, 630-0192, Japan

*Corresponding Author:

miyao@dsc.naist.jp

Section S1 Curation Procedure

Curation of predicted reactants

Predicted reactants up to Top-30 were curated in the following 10 steps.

1. If the predicted reactant was a single compound, it was replaced by NaN.
2. For each reaction (input compound), predicted reactant components were made to a unique set.
3. For each input compound, duplicates in the predicted reactant combinations were excluded.
4. NaN in step 1 was removed.
5. Reactions where the input compound was included in the predicted reactants were excluded.
6. Among the predicted reactants, those that led to the original input compound with the round-trip were retained.
7. Predicted reactants with formal charge were removed.
8. If there were identical combinations of reactants involved in the structure of the same input compound, the more probable reactant combination was retained.
9. If there were combinations where both the replaced reactants and their reaction centers were identical for the same input compound, the more probable reactant combination was retained.
10. Among the remaining predicted reactants up to step 9, the Top-7 predicted reactants with the highest probability were selected.

Curation of extracted compounds from the ZINC database

After the curation of predicted reactants, candidate reactants for the 4,381 replaced reactants were extracted from the ZINC database. After excluding the replaced reactants with no candidate

reactants and then excluding the original replaced reactants from the candidate reactants, 1,922 reactions remained.

Curation of predicted products

Predicted products were curated in the following 4 steps.

1. NaN was removed.
2. Invalid reactions where the product was in the reactants, and products that could not be handled by RDKit, were removed.
3. If there were identical combinations of reactants involved in the structure of the same input compound, the more probable reactant combination in the retrosynthesis prediction was selected.
4. If there were combinations where both the replaced reactants and their reaction centers are identical for the same input compound, the more probable reactant combination in the retrosynthesis prediction was selected.

After the curation of predicted products, 202,798 predicted products remained from the initial 212,516 predicted output.

Table S1. Reaction classes in the training data for training retrosynthesis and forward prediction models. To fine-tune T5Chem, 260,000 reactions were randomly extracted from the reaction database Pistachio, and 210,600 reactions were used as training data.

Reaction class	Count	Percentage [%]
Heteroatom alkylation and arylation	51,156	24.3
Acylation and related processes	42,579	20.2
C-C bond formation	22,682	10.8
Heterocycle formation	7,636	3.6
Protections	4,214	2.0
Deprotections	32,123	15.3
Reductions	12,984	6.2
Oxidations	6,217	3.0
Functional group interconversion	23,374	11.1
Functional group addition	7,635	3.6

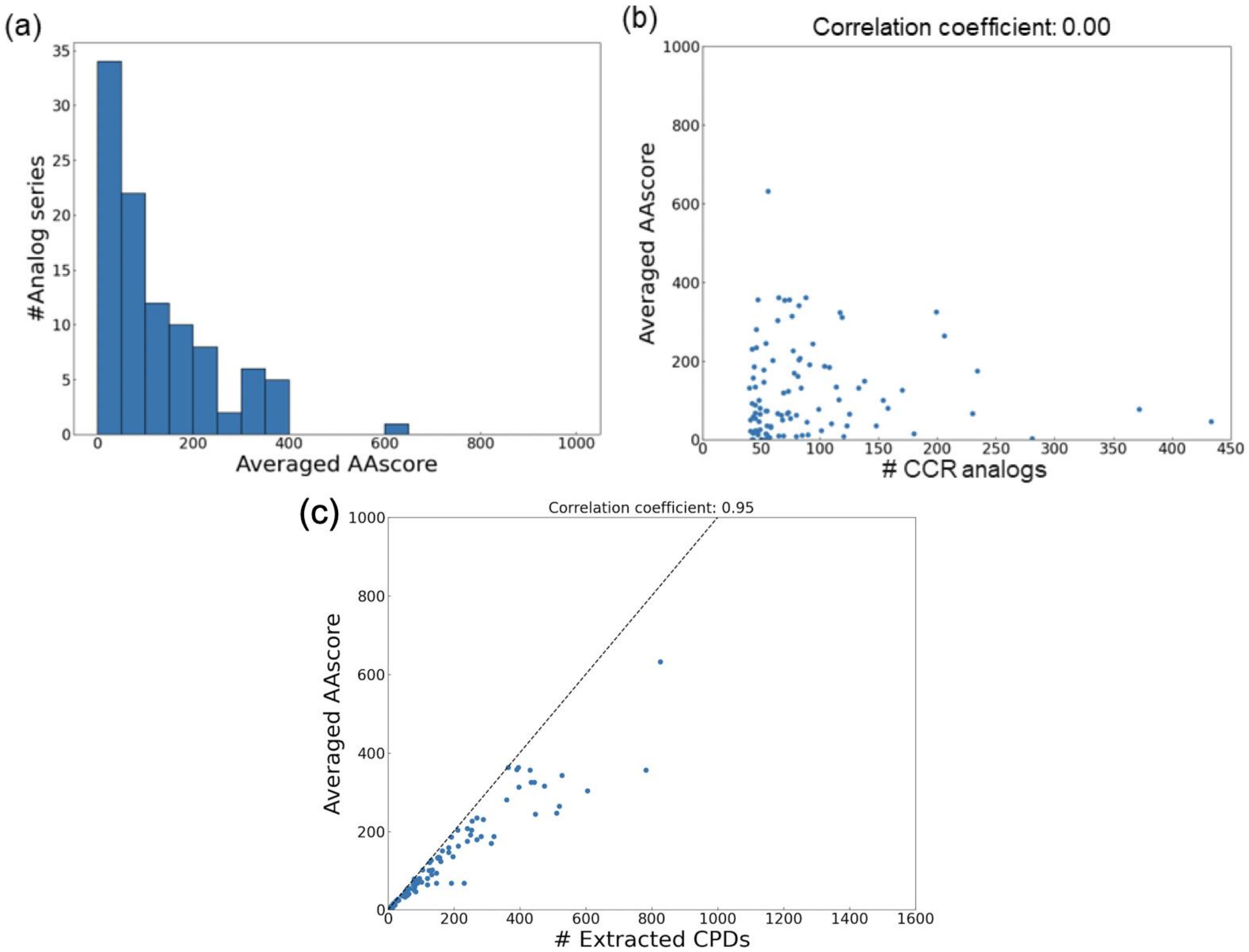

Figure S1. Distribution of the AAscore for the ChEMBL analog series using the ZINC database for searching candidate reactants (retrosynthesis prediction model: LocalRetro). For each analog series, the average of the AAscore for series compounds is assigned. The distribution of the AAscore is reported (**a**). This average AAscore is also plotted against the number of analogs for the core (**b**) and the number of extracted compounds from the ZINC database (**c**), respectively.

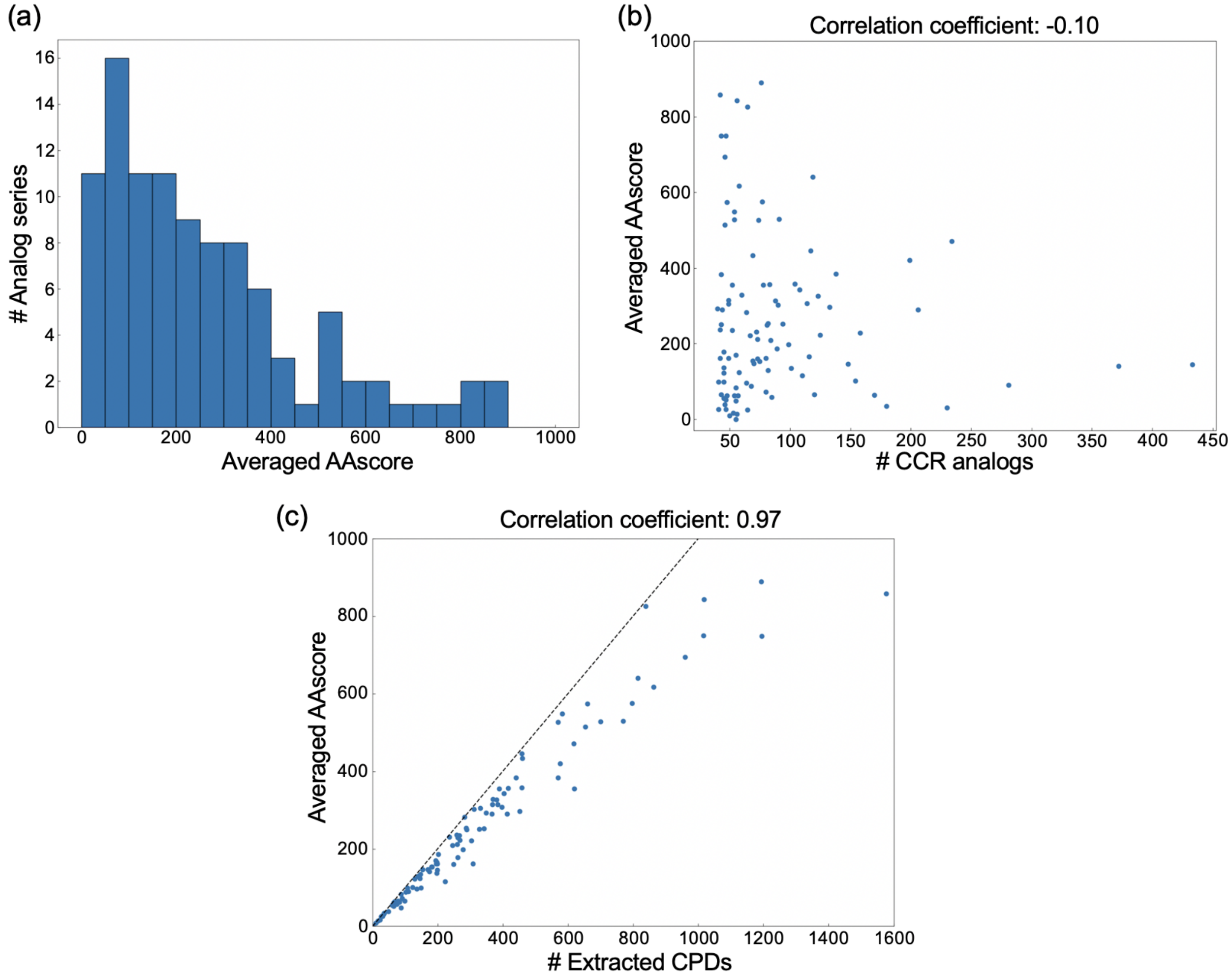

Figure S2. Distribution of the AAscore for the ChEMBL analog series using the eMolecules database for searching candidate reactants. For each analog series, the average of the AAscore for series compounds is assigned. The distribution of the AAscore is reported (**a**). This average AAscore is also plotted against the number of analogs for the core (**b**) and the number of extracted compounds from the eMolecules database (**c**), respectively.

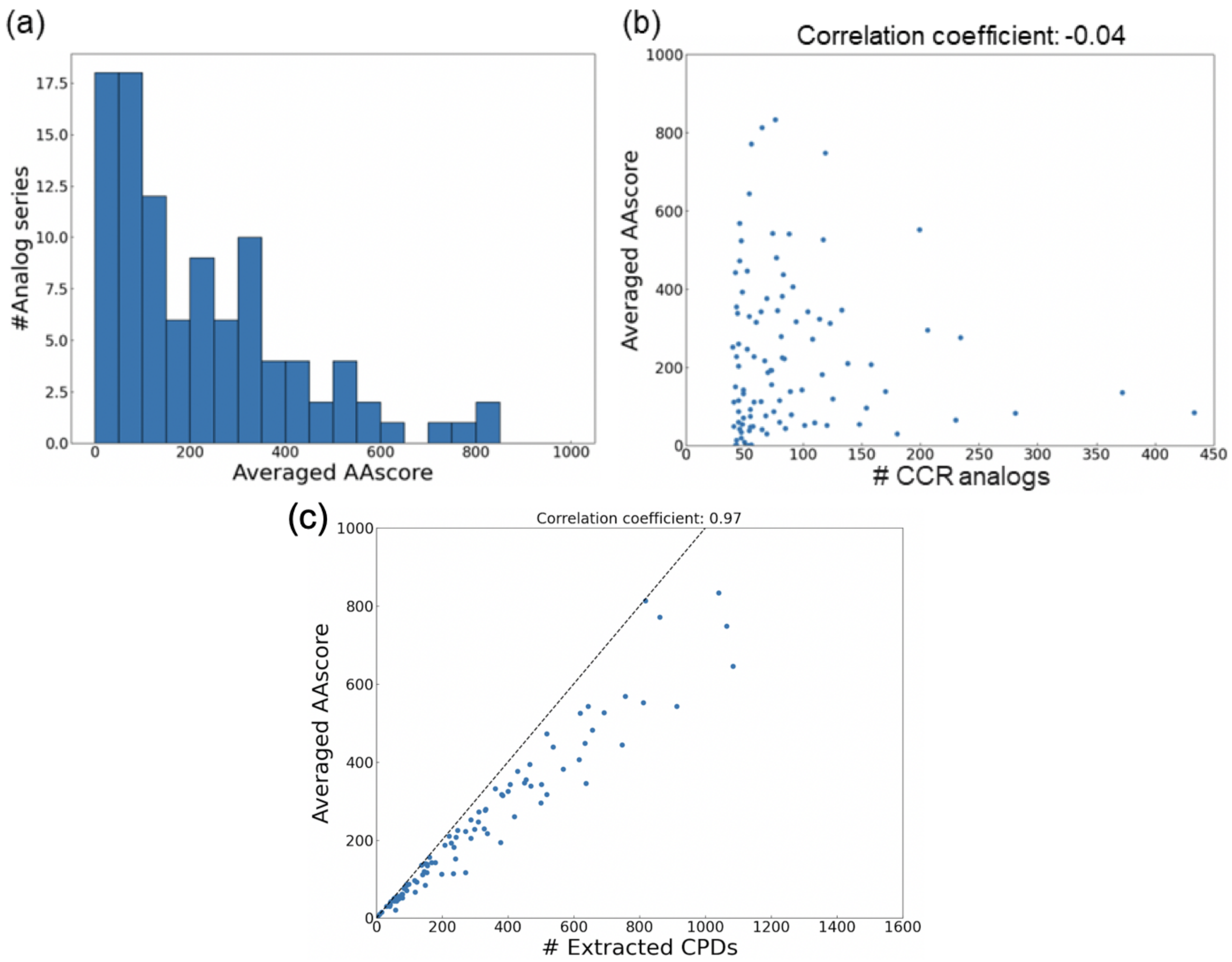

Figure S3. Distribution of the AAscore for the ChEMBL analog series using the eMolecules database for searching candidate reactants (retrosynthesis prediction model: LocalRetro). For each analog series, the average of the AAscore for series compounds is assigned. The distribution of the AAscore is reported (**a**). This average AAscore is also plotted against the number of analogs for the core (**b**) and the number of extracted compounds from the eMolecules database (**c**), respectively.

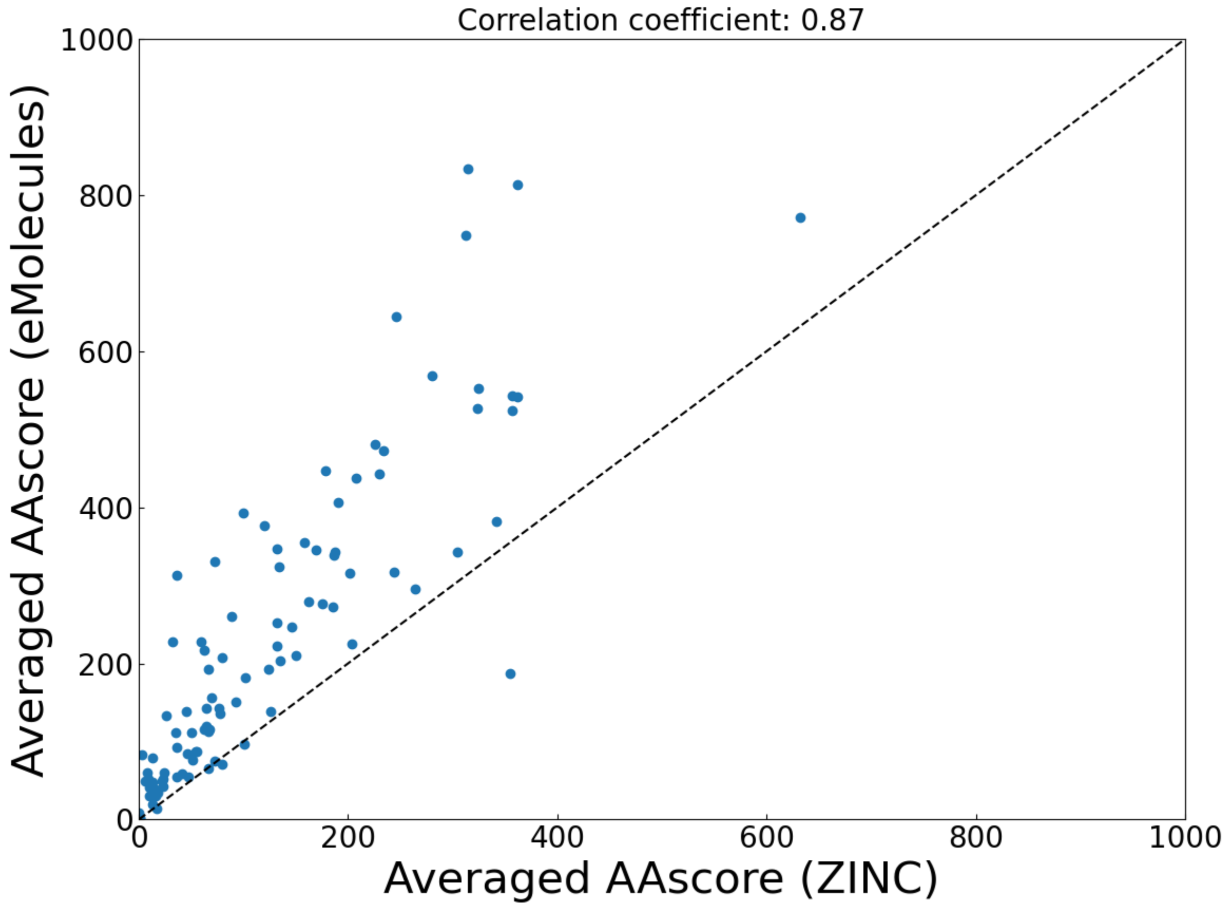

Figure S4. Effect of the databases of purchasable compounds on averaged AAscore (retrosynthesis prediction model: LocalRetro). The averaged AAscore calculated using the eMolecules database is plotted against the averaged AAscore calculated using the ZINC database.
